# Toxicity of Cocaine and Novocaine in Man*Extract from an article published in full in the June *Journal of the N. D. A*.

**Published:** 1918-07

**Authors:** Geo. B. Roth


### TOXICITY OF COCAINE AND NOVOCAINE IN MAN *

BY GEO. B. ROTH, M.D.

It is impossible to determine accurately the relative susceptibility of man to cocaine and novocaine, on account of the fact that the cases of novocaine poisoning are just beginning to find their way into the literature. However, from physiological tests on man and from the available reports of the cases of poisoning an approximate idea of the relative toxicity in man may be computed.

* Extract from an article published in full in the June *Journal of the N. D. A.*

In reviewing the literature relating to cocaine poisoning, it is found that the fatal dose varies from 0.020-0.030 gm. to more than 0.25 gm., while 0.008 gm. may produce dangerous toxic symptoms. Cushny, 1915, states that, for subcutaneous injection, the dose of cocaine should never extend 0.020 gm.

The maximum dose of novocaine tolerated by man without producing general symptoms, like other local anesthetics, depends largely upon the method of its administration. The dosage used by clinicians is remarkably wide. Under certain conditions 0.020 gm. has caused death; where as 2 gms. have been given in other cases without producing symptoms. It would appear, however, that when given intraspinally or for dental operations it may be especially toxic. By the intraspinal method of administration 0.100 gram has produced death.

### CONSIDERATION OF LABORATORY FINDINGS AND THEIR RELATION TO CLINICAL REPORTS

The results of the laboratory experiments with cocaine and novocaine, when compared with the results obtained in the clinical use of these substances, show that man is relatively more susceptible to cocaine and novocaine than are laboratory animals. From animal experiments it is seen that the toxicity of these substances depends partly upon the manner and method of administration. The statement also seems to hold true for man. The untoward results that have been reported in the literature from the use of novocaine in operations about the head and face might well be accounted for by the fact that absorption may be very rapid, as for example in dental operations when the injection is made in a region well supplied with blood vessels, so that administration directly into the circulation is not unlikely to occur, and when injected in this way the toxicity is greater than when given subcutaneously. Individual susceptibility is marked in both animals and man. This may account for some of the fatalities reported in the literature.

### FACTORS WHICH SHOULD BE CONSIDERED IN USE OF COCAINE OR NOVOCAINE

In addition to idiosyncrasy, age seems to be a factor in man in the production of a fatal result with novocaine. In three cases reported by Scandola, 1915, the ages of the men were 69, 75 and 80 years, respectively. In cases having low blood pressure, or cardiac disease, novocaine should be used with caution, inasmuch as in the laboratory experiments it has been shown to have a depressing effect upon the heart muscle when large doses are given.

The administration of hyoscine, previous to the use of a local anesthetic agent, is sometimes advised. If it is given before either cocaine or novocaine, it may act as a synergistic agent in depressing the respiration. In order to prevent the absorption of novocaine from the subcutaneous tissues, epinephrine is employed. Epinephrine is a relatively unstable agent, especially in alkaline solutions. It is not unlikely, therefore, that unless the epinephrine which is used with novocaine is active, general symptoms may arise from the administration of novocaine as a local anesthetic agent.

### CONSIDERATION OF SOME OF THE REPUTED ADVANTAGES OF NOVOCAINE OVER COCAINE

It was previously stated that the widespread use of novocaine has been due largely to the fact that novocaine is considered to be comparatively non-toxic and that it can be sterilized by heat without losing its effectiveness. In addition novocaine is considered to be non-irritant to the tissues, or less irritant than cocaine when used locally.

In searching the literature no reference was found which contradicted the numerous statements which are made indicating that novocaine can be safely sterilized by ordinary means. However, the references relating to the sterilization of cocaine solutions by heat are contradictory. Aqueous solutions of cocaine are usually stated to be unstable and that when boiled undergo decomposition readily. Regarding this point Braun, 1914, states that a single boiling of a solution of cocaine is not followed by a material loss of cocaine. He further states that Tuffier advises the fractional sterilization of cocaine solutions at a temperature of 60 to 70 degrees C. The exhaustive work of Lesure, 1909, demonstrated that solutions of cocaine hydrochloride may be heated in an autoclave at 120 degrees C. for thirty minutes without much loss in activity and that the amount of decomposition varies directly with the alkalinity of the glass. Decomposition was almost negligible when solutions of cocaine were heated in glass containers of low alkalinity while in the ordinary glass containers the loss may not exceed two per cent. When sterilized on the water bath at 100 degrees C. the decomposition was exceedingly small. Lesure concludes that solutions of cocaine hydrochloride may be sterilized at 110-120 degrees C. in closed glass containers provided the alkalinity does not exceed three cubic centimeters of a one-hundredth normal solution of sodium hydroxide. More recently, Holbrook, 1912, found that cocaine hydrochloride was not decomposed to an appreciable extent by boiling for a brief period.

As to the relative amounts of local irritation produced by cocaine and novocaine it may be said that both substances are relatively non-irritant when properly used. Braun, 1914, says that the reports of local damage to the tissues from subcutaneous or submucous injections of cocaine solutions exist only in the older literature and that up to the present time injury to the tissues has never been observed following the injection of dilute cocaine solutions. Dilute solutions of cocaine, up to one per cent., will cause swelling of the tissues and pain. These effects are due to the fact that the solutions are hypotonic. To prevent this sodium chloride an indifferent salt must be added to make them isotonic with the blood.

Many are averse to using cocaine on account of the danger of the patient later becoming a habitue. As yet no report of the occurrence of a novocaine habitue has been made so that it would seem that novocaine is without habit-forming properties; if this be true novocaine possesses a decided advantage over cocaine in this respect.

### SUMMARY AND CONCLUSIONS

1. Novocaine is several times less toxic for laboratory animals than cocaine, the relative toxicity being dependent upon the method of administration as well as upon the animal used in making the determination.
2. Novocaine possesses many of the properties of cocaine as shown by experiments on the isolated heart, on smooth muscle, and by its effects on the circulation and respiration of anesthetized animals.
3. The depressing effect of novocaine on the blood pressure and respiration of animals, makes it necessary to use caution in its administration in clinical cases, in which the blood pressure is low or in which the heart is depressed.
4. Great care should be exercised in the injection of novocaine subcutaneously, in order to avoid its entrance into the circulation thereby increasing its toxicity.
5. Individual susceptibility should always be considered in the administration of either cocaine or novocaine.

